# The Role of *N*^6^-methyladenosine Modification in Gametogenesis and Embryogenesis: Impact on Fertility

**DOI:** 10.1093/gpbjnl/qzae050

**Published:** 2024-06-27

**Authors:** Yujie Wang, Chen Yang, Hanxiao Sun, Hui Jiang, Pin Zhang, Yue Huang, Zhenran Liu, Yaru Yu, Zuying Xu, Huifen Xiang, Chengqi Yi

**Affiliations:** Department of Obstetrics and Gynecology, the First Affiliated Hospital of Anhui Medical University, Hefei 230022, China; NHC Key Laboratory of Study on Abnormal Gametes and Reproductive Tract (Anhui Medical University), Hefei 230032, China; MOE Key Laboratory of Population Health Across Life Cycle (Anhui Medical University), Hefei 230032, China; Department of Obstetrics and Gynecology, the First Affiliated Hospital of Anhui Medical University, Hefei 230022, China; NHC Key Laboratory of Study on Abnormal Gametes and Reproductive Tract (Anhui Medical University), Hefei 230032, China; MOE Key Laboratory of Population Health Across Life Cycle (Anhui Medical University), Hefei 230032, China; State Key Laboratory of Protein and Plant Gene Research, School of Life Sciences, Peking University, Beijing 100871, China; Department of Interventional Therapy, the Second Affiliated Hospital of Anhui Medical University, Hefei 230601, China; Department of Obstetrics and Gynecology, the First Affiliated Hospital of Anhui Medical University, Hefei 230022, China; NHC Key Laboratory of Study on Abnormal Gametes and Reproductive Tract (Anhui Medical University), Hefei 230032, China; MOE Key Laboratory of Population Health Across Life Cycle (Anhui Medical University), Hefei 230032, China; Department of Obstetrics and Gynecology, the First Affiliated Hospital of Anhui Medical University, Hefei 230022, China; NHC Key Laboratory of Study on Abnormal Gametes and Reproductive Tract (Anhui Medical University), Hefei 230032, China; MOE Key Laboratory of Population Health Across Life Cycle (Anhui Medical University), Hefei 230032, China; Department of Obstetrics and Gynecology, the First Affiliated Hospital of Anhui Medical University, Hefei 230022, China; NHC Key Laboratory of Study on Abnormal Gametes and Reproductive Tract (Anhui Medical University), Hefei 230032, China; MOE Key Laboratory of Population Health Across Life Cycle (Anhui Medical University), Hefei 230032, China; Department of Obstetrics and Gynecology, the First Affiliated Hospital of Anhui Medical University, Hefei 230022, China; NHC Key Laboratory of Study on Abnormal Gametes and Reproductive Tract (Anhui Medical University), Hefei 230032, China; MOE Key Laboratory of Population Health Across Life Cycle (Anhui Medical University), Hefei 230032, China; Department of Obstetrics and Gynecology, the First Affiliated Hospital of Anhui Medical University, Hefei 230022, China; NHC Key Laboratory of Study on Abnormal Gametes and Reproductive Tract (Anhui Medical University), Hefei 230032, China; MOE Key Laboratory of Population Health Across Life Cycle (Anhui Medical University), Hefei 230032, China; Department of Obstetrics and Gynecology, the First Affiliated Hospital of Anhui Medical University, Hefei 230022, China; NHC Key Laboratory of Study on Abnormal Gametes and Reproductive Tract (Anhui Medical University), Hefei 230032, China; MOE Key Laboratory of Population Health Across Life Cycle (Anhui Medical University), Hefei 230032, China; State Key Laboratory of Protein and Plant Gene Research, School of Life Sciences, Peking University, Beijing 100871, China; Peking-Tsinghua Center for Life Sciences, Peking University, Beijing 100871, China; Department of Chemical Biology and Synthetic and Functional Biomolecules Center, College of Chemistry and Molecular Engineering, Peking University, Beijing 100871, China

**Keywords:** Spermatogenesis, Oogenesis, Epigenetics, *N* ^6^-methyladenosine, Reproduction

## Abstract

The most common epigenetic modification of messenger RNAs (mRNAs) is *N*^6^-methyladenosine (m^6^A), which is mainly located near the 3′ untranslated region of mRNAs, near the stop codons, and within internal exons. The biological effect of m^6^A is dynamically modulated by methyltransferases (writers), demethylases (erasers), and m^6^A-binding proteins (readers). By controlling post-transcriptional gene expression, m^6^A has a significant impact on numerous biological functions, including RNA transcription, translation, splicing, transport, and degradation. Hence, m^6^A influences various physiological and pathological processes, such as spermatogenesis, oogenesis, embryogenesis, placental function, and human reproductive system diseases. During gametogenesis and embryogenesis, genetic material undergoes significant changes, including epigenomic modifications such as m^6^A. From spermatogenesis and oogenesis to the formation of an oosperm and early embryogenesis, m^6^A changes occur at every step. m^6^A abnormalities can lead to gamete abnormalities, developmental delays, impaired fertilization, and maternal-to-zygotic transition blockage. Both mice and humans with abnormal m^6^A modifications exhibit impaired fertility. In this review, we discuss the dynamic biological effects of m^6^A and its regulators on gamete and embryonic development and review the possible mechanisms of infertility caused by m^6^A changes. We also discuss the drugs currently used to manipulate m^6^A and provide prospects for the prevention and treatment of infertility at the epigenetic level.

## Introduction

Epigenetics is the study of heritable changes that affect gene expression but do not result from DNA [1] or other nucleotide sequence alterations [2], such as DNA methylation, histone modification, chromatin rearrangement, and RNA modification, which are essential for controlling many physiological and pathological processes [3]. More than 160 structurally different RNA modifications have been identified in eukaryotes [4,5]. Methylation of bases and 2′-hydroxylation of RNA nucleotides are the most prevalent and straightforward RNA modifications [6]. *N*^6^-methyladenosine (m^6^A) is the most common form of messenger RNA (mRNA) modifications and was discovered in cancer cells as early as 1974 [7,8]. Each mRNA molecule exhibits, on average, between three and five sites of m^6^A modification [9]. With the advancement of epigenetics and the application of high-throughput sequencing technology, the biological functions and clinical applications of m^6^A have received increasing attention [10]. The biological effect of m^6^A is dynamically modulated by methyltransferases (writers), demethylases (erasers), and m^6^A-binding proteins (readers). Writers, encompassing a multicomponent methyltransferase complex, are responsible for adding m^6^A modifications [11]; erasers can remove m^6^A modifications [12]; and readers can recognize m^6^A modifications that mediate different downstream processes [13]. m^6^A modification typically occurs near exonic or intronic splice junctions and can therefore directly affect splicing [3]. It can modulate post-transcriptional gene expression by regulating pre-mRNA splicing and mRNA export, stability, and translation [14–19].

One of the most tightly controlled biological processes in a eukaryote’s life cycle is sexual reproduction, which is one of the most basic functions [20]. Germ cells encompass a special type of cell that can undergo meiosis. From late meiosis to early embryonic development, there is a period of gene transcriptional inactivity in the process of maternal-to-zygotic transition (MZT), and precise regulation of gene expression relies on post-transcriptional epigenetic modifications [21]. Hence, the m^6^A modification plays a crucial role in the initiation and progression of meiosis as well as in unique processes such as sperm–oocyte interactions and sex determination [22]. For example, defective gamete maturation has been associated with a reduction in the overall amount of m^6^A modifications [23], and only females have survived in the inducers of meiosis 4 (*Ime4*) knockout (KO) fruit flies [22,24]. Impaired parental gamete formation or abnormal embryonic development of offspring will lead to infertility.

In this review, we discuss the dynamic biological effects of the m^6^A modification and its regulators during gametic and embryonic development. In addition, we discuss drugs that can affect this modification and provide insights into strategies for the prevention and treatment of infertility at the epigenetic level.

## m^6^A modification regulators

**Figure 1:**
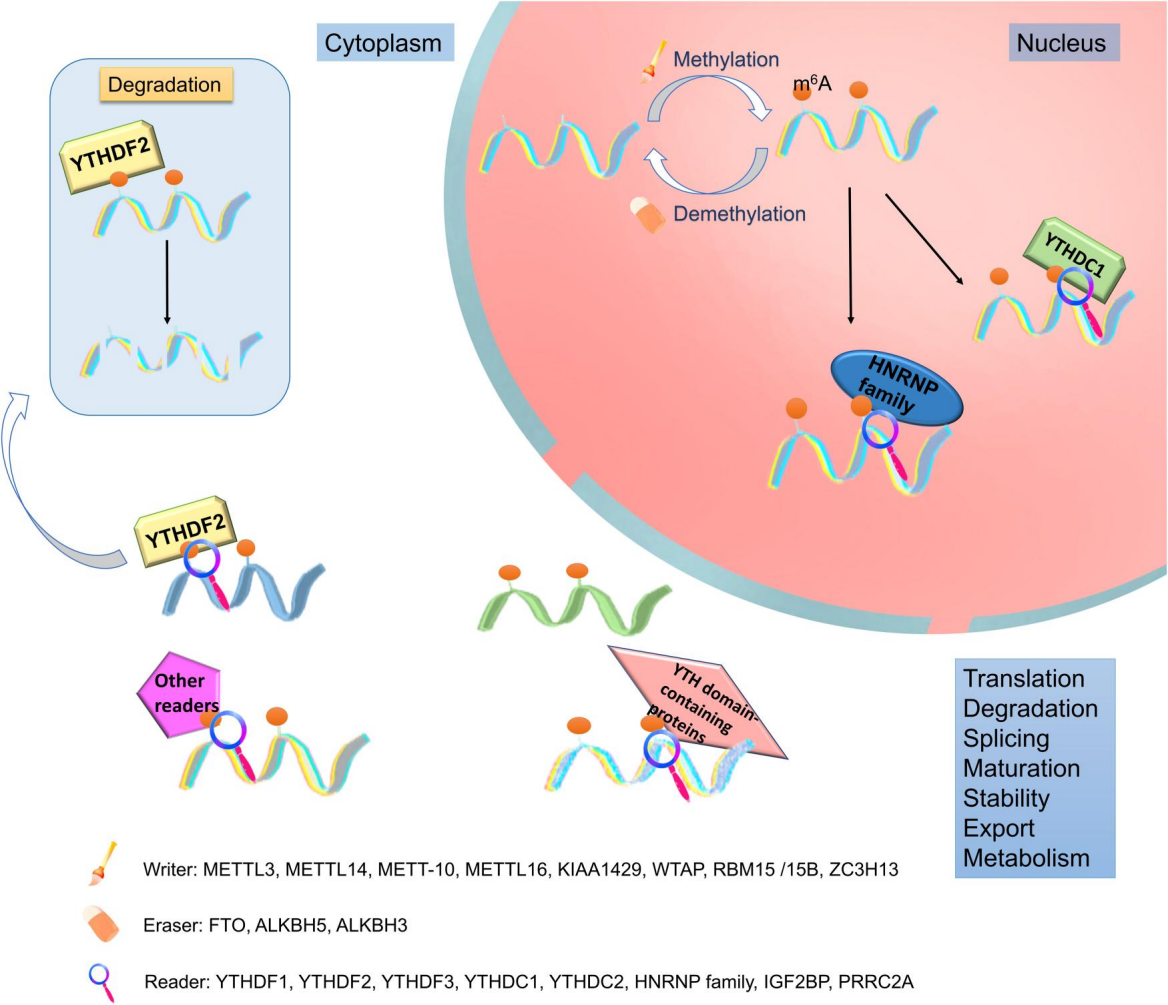
Mechanism and biological function of m^6^A methylation Writers (methyltransferases) are responsible for the catalysis of m^6^A. Demethylases, known as erasers, can mediate methylation reversal by eliminating the m^6^A modification. Readers can recognize m^6^A-modified mRNAs and change downstream outcomes. m^6^A, *N*^6^-methyladenosine; METTL3, methyltransferase-like protein 3; METTL14, methyltransferase-like protein 14; METT-10, a conserved putative methyltransferase; METTL16, methyltransferase-like protein 16; KIAA1429, vir-like m^6^A methyltransferase associated protein; WTAP, Wilms tumor 1-associating protein; RBM15/15B, RNA-binding motif protein 15/15B; ZC3H13, zinc finger CCCH-type containing 13; FTO, fat mass and obesity-associated protein; ALKBH5, alpha-ketoglutarate-dependent dioxygenase homolog 5; ALKBH3, alpha-ketoglutarate-dependent dioxygenase homolog 3; YTHDF1, YTH *N*^6^-methyladenosine RNA-binding protein 1; YTHDF2, YTH *N*^6^-methyladenosine RNA-binding protein 2; YTHDF3, YTH *N*^6^-methyladenosine RNA-binding protein 3; YTHDC1, YTH domain-containing 1; YTHDC2, YTH domain-containing 2; HNRNP, heterogeneous nuclear ribonucleoprotein; IGF2BP, human insulin-like growth factor-2 mRNA-binding protein 2; PRRC2A, proline rich coiled-coil 2A.

The m^6^A modification is mainly concentrated in the 3′ untranslated regions (UTRs) and protein-coding sequences near the mRNA stop codon [16]. In addition to its distinctive distribution, it is characterized as dynamic, reversible, and widespread [12,25]. The addition of m^6^A is catalyzed by methyltransferases; demethylases, known as erasers, can mediate methylation reversal by eliminating the m^6^A modification; and readers are RNA-binding proteins that can recognize the regulatory effect of m^6^A on mRNA and change its downstream effects [14–19], influencing the mRNA’s fate [26,27] (**Figure 1**; **Table 1**).

**Table 1:** Role of m^6^A regulators and biological mechanisms in the reproductive system

Type	Source	Regulator	Function	Refs.
Writer	Mouse, zebrafish, pig	METTL3	Promote embryonic development and cell reprogramming; promote spermatogenesis; affect the sex ratio	[23,82,84–86,91]
	Mouse, human	METTL14	Promote embryonic development and spermatogenesis; synergize with METTL3	[85,87,89,102]
	*Caenorhabditis elegans*	METT-10 (a homolog of METTL16)	Inhibit germ cell proliferation; promote vulva, somatic gonad, and embryonic development	[10,93]
	*Caenorhabditis elegans*, mouse	METTL16	Promote embryonic development; mediate mRNA splicing	[93,121]
	Mouse	KIAA1429	Participate in alternative splicing of genes related to oogenesis	[96,97]
	Pig, mouse	WTAP	Regulate the gene expression of supporting cells at the levels of transcription and translation	[82,88,89]
	Mouse	RBM15/15B	Control RNA splicing; promote XIST-mediated X chromosome silencing	[38,39]
	Mouse	ZC3H13	Promote embryonic development	[41]
Eraser	Pig, mouse, human	FTO	Maintain male fertility; regulate the apoptosis of Leydig cells; promote oocyte maturation; promote embryonic development	[82,89,101–104,127]
	Mouse, human	ALKBH5	Promote meiosis and spermatogenesis; maintain male fertility; inhibit interferon production; promote breast cancer cell proliferation	[45–47,89,98,103]
	Bovine	ALKBH3	Increase protein translation efficiency	[48]
Reader	Mouse, HeLa cell	YTHDF1	Improve translation efficiency; participate in the regulation of BTB integrity	[17,100]
	Zebrafish	Ythdf2	Promote mRNA degradation and maternal-to-zygotic transition	[13,59]
	Zebrafish	Ythdf3	Maintain female gonadal development; likely have synergistic effects with YTHDF1	[13]
	Mouse	YTHDC1	Regulate mRNA splicing and translation; promote oocyte maturation and embryonic development; promote XIST-mediated gene repression	[38,97,105]
	Mouse, FGC	YTHDC2	Affect translation efficiency and mRNA abundance; promote XIST-mediated gene repression	[54,60,61]
	HeLa cell, HEK293T cell	HNRNP	Participate in the alternative splicing of mRNA and the splicing process of miRNA precursors to regulate RNA maturation and affect the abundance of target transcripts	[50]
	HepG2 cell	IGF2BP	Maintain the stability of target RNA and promote the translation process	[56]
	Mouse	PRRC2A	Promote glial cell development; modulate XY synapsis and MSCI	[107,108]

*Note*: METTL3, methyltransferase-like protein 3; METTL14, methyltransferase-like protein 14; METT-10, a conserved putative methyltransferase; METTL16, methyltransferase-like protein 16; KIAA1429, vir-like m^6^A methyltransferase associated protein; WTAP, Wilms tumor 1-associating protein; RBM15/15B, RNA-binding motif protein 15/15B; ZC3H13, zinc finger CCCH-type containing 13; FTO, fat mass and obesity-associated protein; ALKBH5, alpha-ketoglutarate-dependent dioxygenase homolog 5; ALKBH3, alpha-ketoglutarate-dependent dioxygenase homolog 3; YTHDF1/2/3, YTH *N*^6^-methyladenosine RNA-binding protein 1/2/3; YTHDC1/2, YTH domain-containing 1/2; HNRNP, heterogeneous nuclear ribonucleoprotein; IGF2BP, human insulin-like growth factor-2 mRNA-binding protein 2; PRRC2A, proline rich coiled-coil 2A; BTB, blood‒testis barrier; XIST, X-inactive specific transcript; MSCI, meiotic sex chromosome inactivation; FGC, female germ cell; mRNA, messenger RNA; miRNA, microRNA; m^6^A, *N*^6^-methyladenosine.

### Writers (m^6^A methyltransferases)

Methyltransferase complexes consist of two subcomplexes and catalyze the addition of m^6^A modification. They include Wilms tumor 1-associating protein (WTAP), methyltransferase-like protein 3 (METTL3), methyltransferase-like protein 14 (METTL14), and vir-like m6A methyltransferase associated protein (KIAA1429) [28]. Other members include RNA-binding motif protein 15 (RBM15) and its homolog, RBM15B, and zinc finger CCCH-type containing 13 (ZC3H13), which are in charge of converting the donor *S*-adenosylmethionine (SAM) to adenine by transferring methyl groups [26,29–31]. SAM, which functions as the methyl donor for the production of m^6^A, undergoes methylation of either nitrogen or oxygen atoms at the post-transcriptional level [32,33]. In 1997, METTL3 was identified as an important catalyst of m^6^A modification [34]. Both the stability of mRNA and the level of m^6^A methylation can be directly impacted by METTL3 expression [35]. By forming a heterodimer complex with METTL14, METTL3 can detect the substrate and play an active role [35,36]. KIAA1429 and RBM15 maintain the m^6^A levels by recruiting methyltransferase complexes to specific RNA regions [37–39]. WTAP contributes to alternative splicing, mRNA stabilization, and cell growth [40]. Knockdown (KD) of *Zc3h13* in mouse embryonic stem cells reduces global polyadenylated RNA m^6^A levels which occurs predominantly in the 3′ UTR [41].

### Erasers (m^6^A demethylases)

Erasers are demethylating enzymes that include fat mass and obesity-associated protein (FTO), human alpha-ketoglutarate-dependent dioxygenase (AlkB) homolog H5 (ALKBH5), and ALKBH3. ALKBH5 and FTO, respectively, can remove m^6^A modifications in ferrous- and α-ketoglutaric acid-dependent ways [27,42,43]. FTO was the first demethylase discovered [44] and showed that m^6^A modification is dynamically regulated. The second demethylase, ALKBH5, affects the proper assembly/modification of mRNA processing factors [45–47]. The novel demethylase ALKBH3 appears to enhance the effectiveness of protein translation by demethylating transfer RNA (tRNA) [48], but it acts on *N*^6^-methyladenine instead of *N*^6^-methyladenosine.

### Readers (m^6^A-binding proteins)

m^6^A modification can be recognized by various RNA-binding proteins known as readers, including YTH domain-containing proteins [YTH *N*^6^-methyladenosine RNA-binding protein F1/2/3 (YTHDF1/2/3) and YTH domain-containing 1/2 (YTHDC1/2)], proline rich coiled-coil 2A (PRRC2A), insulin-like growth factor-2 mRNA-binding proteins (IGF2BP1/2/3), and heterogeneous nuclear ribonucleoprotein (HNRNP) family proteins [14,49–51]. The effect of m^6^A modification mainly depends on downstream RNA-binding proteins, which prioritize m^6^A-modified RNA recognition and integrate m^6^A methylation with RNA processing and other biological processes [52,53]. They mediate multiple molecular functions, including RNA splicing, mRNA abundance, and translation efficiency [54]. IGF2BP2 plays a role in the occurrence and progression of cancer through communication with different RNAs and can maintain the stability of target RNA and promote the translation process [55,56]. YTHDF1 can improve translation efficiency, and YTHDF3 may synergize with YTHDF1 [13,17]. The nuclear m^6^A reader YTHDC1 controls nuclear RNA export, participates in alternative splicing, and promotes oocyte maturation and embryo maturation [57,58]. YTHDF2 can promote mRNA degradation and has minimal effects on its stability [13,59]. YTHDC2 affects translation efficiency and mRNA abundance, and promotes X-inactive specific transcript (XIST)-mediated gene repression [54,60,61]. HNRNPA2B1 recognizes m^6^A-modified RNA using a nonspecific (m^6^A consensus motif is short) method to enhance the processing and production of METTL3*-*dependent tiny RNAs [14,62].

## m^6^A modification and infertility

Infertility is defined as a failed pregnancy following 12 months of therapeutic donor insemination or proper, scheduled unprotected sexual activity. For women over 35 years of age, examination and treatment are recommended after 6 months [63,64]. Changes in epigenetic modifications in spermatozoa lead to a significant increase in DNA damage after the age of 40 years old [65].

**Figure 2:**
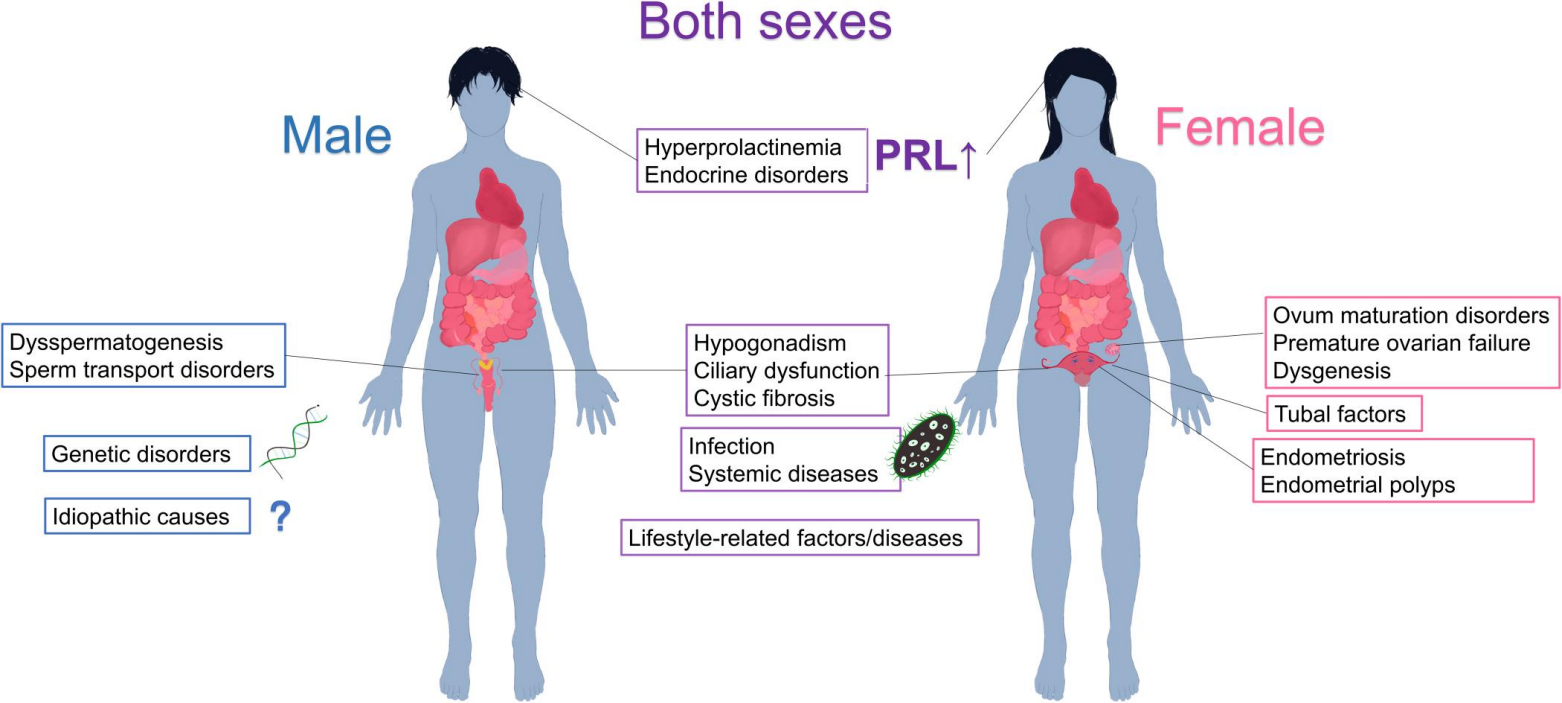
Main causes of infertility The causes of infertility can be organic or social factors. PRL, prolactin.

According to demographic studies, the birth rate has steadily declined in all European countries since the early 1950s, with similar trends across regions and social classes [66]. Infertility has progressively increased in prevalence and incidence globally, becoming an important issue. Approximately 15% of couples (48.5 million people) worldwide are affected by infertility [67], with the incidence being higher in developing countries [68]. More than half of all cases are caused by male infertility [68]. Compared to oocytes, spermatocytes undergo more frequent division and differentiation, which can lead to epigenetic modifications and abnormal sperm production [69,70]. Male infertility is mostly caused by dysspermatogenesis, sperm transport/genetic disorders, and idiopathic causes [71]. Factors leading to female infertility include premature ovarian failure, ovum maturation disorders, dysgenesis, and tubal and endometrial factors. Factors affecting fertility in both sexes include hypogonadism, hyperprolactinemia, ciliary dysfunction, cystic fibrosis, infection, endocrine disorders, systemic disorders, and lifestyle-related conditions [72] (**Figure 2**). Infertility can be overcome through prevention, diagnosis, and early treatment. The best approach to treating infertility caused by gamete abnormalities is preimplantation genetic testing. Improving economic and educational levels is a recommended prevention strategy [73], and new drugs for treatment are urgently needed.

m^6^A is essential for numerous physiological and pathological processes, and the reproductive system is no exception. Abnormal m^6^A modifications lead to gamete formation disorders and abnormal embryonic development, thereby affecting fertility.

## m^6^A in gametogenesis

**Figure 3:**
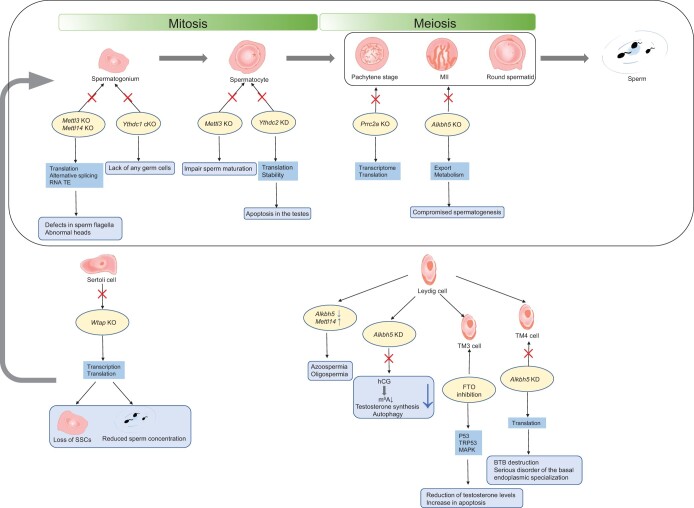
Effects of changes in m^6^A regulators on male germ cells The role of m^6^A and its regulators in different stages of spermatogenesis, including spermatogenic cells and testicular somatic cells. The m^6^A modification-related proteins exert stage-dependent functions during spermatogenesis in mammals. MII, metaphase II; KO, knockout; KD, knockdown; SSC, spermatogonial stem cell; TE, transposable element; TM3, mouse Leydig cell; TM4, mouse testicular Sertoli cell; hCG, human chorionic gonadotropin.

Meiosis, a process of reduced division known as gametogenesis, is necessary for the development of viable gametes in mammals. Sperm production is an orderly, accurate, and complex process [69,74,75] that includes the following three regulated and continuous steps: spermatogonia undergo mitosis and differentiate into spermatocytes; meiosis follows to form haploid spermatids; and the haploid spermatids finally transform into spermatozoa [76]. During mitosis, spermatogonial stem cells (SSCs) divide into spermatogonia that are A-paired (Apr) and A-aligned (Aal). Aal spermatogonia can divide and proliferate again to form Aal spermatogonia, and A1 spermatogonia can irreversibly differentiate into type B spermatogonia. B spermatogonia enter meiosis as primary spermatocytes after mitosis. By the end of the first meiosis, each primary spermatocyte will have formed two secondary spermatocytes, which yield two haploid spermatids at the end of the second meiosis [69,75] (**Figure 3**).

**Figure 4:**
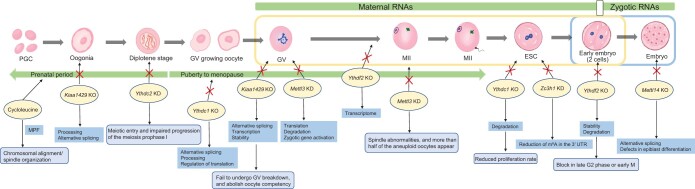
Effects of changes in m^6^A regulators on female germ cells and embryos The role of m^6^A and its regulators in different stages of oogenesis and embryogenesis. The m^6^A level gradually decreases from the GV stage to the two-cell stage, with a gradual decrease in the accumulation of maternal RNAs in the cytoplasm. After the ZGA of embryos during cleavage, an increase in cytoplasmic RNAs is accompanied by an increase in m^6^A signaling from the two-cell stage to the blastocyst stage. ZGA, zygotic genome activation; PGC, primordial germ cell; MPF, maturation-promoting factor; UTR, untranslated region; GV, germinal vesicle; ESC, embryonic stem cell.

Oogenesis in mammals is a protracted process that starts during the embryonic stage and culminates in menopause. Oocyte development involves oogenesis, folliculogenesis, and ovulation. Primordial germ cells (PGCs) undergo mitosis to produce oogonia, which undergo mitosis again and enter meiosis to produce primary oocytes. After the first meiosis, a secondary oocyte and the first polar body are formed. Secondary oocytes enter the second meiotic stage to form egg cells and secondary polar bodies [21] (**Figure 4**).

m^6^A modification is key to this complex and orderly process, as any abnormality at any step interrupts spermatogenesis and oogenesis. In mammalian oocytes, after active transcription during the rapid development stage, germinal vesicles (GVs) in the nucleus progressively become inactive [77]. Early embryonic development and meiosis completion depend largely on oocyte-derived maternal RNAs in the absence of active transcription [21]. A study of *Xenopus laevis* compared m^6^A levels in GVs and metaphase II (MII) oocytes and found that m^6^A levels decreased mostly from the GV stage to the MII stage [78]. Similarly, the m^6^A content in mouse GVs was higher than that in MII oocytes [79]. In a study of porcine granulosa cells, the presence of m^6^A modifications gradually decreased during the growth of small follicles (< 3 mm) into large follicles (> 5 mm) [80]. The overall negative connection between m^6^A methylation and gene expression suggests that follicle selection may be influenced by the timing and location of m^6^A peaks in various follicles [81]. However, elevated levels of endogenous m^6^A have been observed during the meiosis of porcine oocytes [82]. These results indicate that the m^6^A distribution is species-specific [80]. Research on the m^6^A landscape has revealed that species, not tissue type, is the main factor influencing the methylome [83].

### m^6^A writers in gametogenesis

Male fertility and cell differentiation during spermatogenesis depend on METTL3. According to a prior study, *Mettl3* conditional knockout (cKO) mice do not have pachytene-stage spermatocytes [84]. METTL3 and METTL14 act synergistically [85], and their deletion leads to impaired spermiogenesis. The loss of m^6^A results in the depletion of SSCs when *Mettl3* and *Mettl14* are specifically knocked out in germ cells, and the KO of either gene produces an imbalance in the translation of genes enriched with m^6^A in SSCs [86]. In humans, high-level m^6^A can impede sperm motility, according to a study on asthenozoospermia, which also reveals that METTL3 plays an important role in the dynamic modulation of m^6^A in patients with this condition. The mRNA expression of *METTL3* and *METTL14* was higher in the asthenozoospermia group than that in the control group [87]. During meiosis in zebrafish, sperm maturation was inhibited in a *mettl3* KO line (zygotic *mettl3* mutant). In the early stages of spermatogenesis, spermatogonia and spermatocytes in the lobular cavities were smaller, with very little or no mature sperm in the testes of males in the KO lines [23].

Along with testicular somatic cells, Sertoli and Leydig cells are essential for gamete development. When WTAP, the main component of the m^6^A methyltransferase complex, is deleted in mouse Sertoli cells, a gradual loss of SSCs occurs with a reduction in the number of germ cells [88]. In Leydig cells with azoospermia or oligospermia, the expression levels of *Mettl14* and *Alkbh5* were elevated and decreased, respectively [89]. However according to another study, METTL3, not ALKBH5, is crucial for human male infertility, particularly in the case of asthenozoospermia [87].

When primordial follicles become primary follicles, gene expression significantly changes [90]. Similarly, abnormal epigenetic modifications during oogenesis can lead to abnormal oocyte development. In mice, *Mettl3* KD oocytes during MII period showed obvious spindle abnormalities, with a much higher rate of aneuploidy than that in the control group [91]. In *Mettl3* KO females, oocyte maturation was disturbed in the early stages of oogenesis. The ratio of full-grown-stage follicles was much lower than that of wild-type follicles, and the majority of oocytes were arrested in the early full-growth, previtellogenic, early vitellogenic, or mid-vitellogenic stage [23]. In cultivated *Bombyx mori* cells, the KD of *BmMettl3* and *BmMettl14* resulted in the stoppage of cell cycle development and a deficit in chromosome alignment and segregation [92]. In *Caenorhabditis elegans*, METT-10 (a homolog of METTL16) inhibited germ cell proliferation [93]. In *Saccharomyces cerevisiae*, *IME4* is a crucial gene in a tightly controlled system that results in meiosis and sporulation [94]. When *IME4* expression was enhanced during yeast spore production, m^6^A modifications in the overall RNA increased 1.6-fold at the same time [95]. KIAA1429 is a protein of the methyltransferase complex. In GV oocytes with widespread transcriptome abnormalities, *Kiaa1429* KO drastically reduced the quantity of m^6^A transcripts [96]. GV breakdown is an important step in oogenesis and marks oocyte maturity. Oocyte-specific *Kiaa1429* KO resulted in female infertility and follicular dysplasia, primarily affecting the alternative splicing of oogenesis-related genes, and fully developed GV oocytes failed to undergo GV breakdown [97].

### m^6^A erasers in gametogenesis

The global inactivation of *Alkbh5* (in *Alkbh5* KO mice) did not result in observable developmental abnormalities or adult illnesses, except for male sterility [45]. *Alkbh5* KD male mice had elevated mRNA m^6^A levels and decreased fertility due to the apoptosis of spermatocytes in the meiotic metaphase stage [45]. ALKBH5 is required for the late meiotic and haploid phases of spermatogenesis, and mid-pachytene spermatocytes or round spermatids cannot be detected in *Alkbh5* KO mice at a specific developmental stage [98].

The blood–testis barrier (BTB) forms and maintains a microenvironment that is favorable to spermatogenesis, prevents spermatogenic substances from escaping from the spermatogenic tubules to trigger autoimmune reactions, and prevents certain substances from entering the spermatogenic epithelium [99]. ALKBH5 in Sertoli cells is key to the BTB, and basal endoplasmic specialization is severely disrupted in the testicles of *Alkbh5* KO mice [100]. Testosterone is an important sex hormone in both males and females, the synthesis of which is associated with autophagy. m^6^A methylation regulates autophagy in Leydig cells, which control the production of testosterone [89]. KD of *ALKBH5*, but not *FTO*, significantly inhibited human chorionic gonadotropin (hCG)-induced m^6^A reduction, testosterone production, and autophagy in Leydig cells. Additionally, m^6^A and FTO could be related to apoptosis in Leydig cells caused by environmental toxins [101,102]. In mice, Leydig cells show a gradual increase in the mRNA levels of *Alkbh5* and *Fto* during development, but not significantly changing levels of *Mettl3*, *Mettl14*, or *Wtap* [89]. Reduced semen quality is highly correlated with genetic alterations in human *FTO*, and abnormal mRNA demethylation poses a risk for reduced male fertility [103]. Missense mutations in *FTO* and *ALKBH5* have been identified in men who have undergone infertility testing [103], showing that m^6^A is related to abnormalities in male sperm and infertility. *Fto* KO female mice displayed ovarian malformations and decreased fertility [104]. However, studies of m^6^A erasers in oogenesis are limited.

### m^6^A readers in gametogenesis

Reader proteins play important roles in spermatocyte division overshoot. YTHDC1 is a reader protein that regulates mRNA splicing and translation. In their initial developmental period, although grossly normal and alive, *Ythdc1* cKO mice lacked germ cells, including mitotic spermatogonia, and had a Sertoli cell-only phenotype similar to that of adults [105]. This suggests that the loss of *Ythdc1* does not cause death in mice but causes male sterility. The inactivation of *Ythdc1* prevents oocyte maturation, leading to female sterility [105]. YTHDC2 is another m^6^A reader protein that influences the translation efficiency and mRNA abundance of its targets. In *Ythdc2* KO mice, changes in the mRNA expression of *Smc3* [a top RNA immunoprecipitation sequencing (RIP-seq) target of Ythdc2 involved in spermatogenesis] and *Cep76* (a main RIP-seq target of Ythdc2 involved in centriole reduplication), which affect translational efficiency, led to a reduction in spermatocytes [54]. While *Ythdf2* KO females were infertile and had corpora lutea in their ovaries, indicating that ovulation has taken place but has resulted in female-specific infertility, *Ythdf2* KO males were fertile and had normal seminiferous tubule histology [106]. Another reader protein, PRRC2A, is associated with sex chromosomes, as the specific *Prrc2a* KO in spermatocytes causes XY asynapsis, resulting in chromosomal and spindle disorders during meiosis [107,108]. In zebrafish, the *ythdf2* and *ythdf3* double mutations impaired female gonad function [13].

In both mice and humans, writers, erasers, and readers play important roles in spermatogenesis, and any abnormal m^6^A modifications in these processes eventually lead to abnormal semen production, resulting in infertility (**Table 2**). RNA m^6^A modification regulates spermatogenesis in almost all types of spermatogenic and testicular somatic cells, and abnormalities in these cells lead to abnormal semen and infertility, which is the only clinical symptom of male infertility. A growing number of studies have indicated that m^6^A modification is crucial for numerous physiological and pathological processes in the female reproductive system, thus affecting female fertility (**Table 3**). At present, it is only known that m^6^A modification plays a role in oocyte meiosis, and its exact role in oocyte development at different embryonic stages remains unclear.

**Table 2:** Role of m^6^A and its regulators in spermatogenesis

Period of occurrence	Regulator	Species	m^6^A perturbation	Phenotype	mRNA biology	Refs.
Undifferentiated spermatogonium	METTL3, METTL14	Mouse	*Mettl3* cKO, *Mettl14* cKO (Stra8)	Impair spermatogenesis (*e.g.*, defect in sperm flagella and abnormal heads)	Translation, alternative splicing, and translational efficiency	[84,86]
Spermatogonium	YTHDC1	Mouse	*Ythdc1* KO (DDX4)	Lack of any germ cells		[105]
Spermatogonium and spermatocyte	Mettl3	Zebrafish	*mettl3* KO (TALENs)	Little or no mature sperm cells in the testes		[23]
Spermatocyte	YTHDC2	Mouse	*Ythdc2* KD	Apoptosis in the testes	Translation and stability	[54]
Metaphase-stage spermatocyte	PRRC2A	Mouse	*Prrc2a* KO	Cause XY asynapsis, MSCI, chromosomal disorders, and spindle disorders	Translation	[107]
Pachytene-stage spermatocyte, metaphase-stage spermatocyte, spermatocyte, and round spermatid	ALKBH5	Mouse	*Alkbh5* KO	Compromise spermatogenesis	Export and metabolism	[45,98]
TM3 cell	FTO	Leydig cells (mouse)	FTO inhibition	Reduce testosterone level and increase apoptosis	P53, TRP53, and MAPK signaling pathways	[102]
Sertoli cell	WTAP	Mouse	*Wtap* KO	Reduce sperm cell concentration	Transcription and translation	[88]
Sertoli cell (TM4)	ALKBH5	Mouse	*Alkbh5* KO	BTB destruction and serious disorder of the basal endoplasmic specialization	Translation	[100]
Primary Leydig cell and TM3 cell	Autophagy	Leydig cells (mouse)	hCG	Testosterone synthesis increase	Stability, translation, and AMPK signaling pathway	[89]
Human semen sample	METTL3, METTL14	Human	*METTL3* and *METTL14* up-regulation	Asthenozoospermia		[87]

*Note*: KO, knockout; cKO, conditional knockout; KD, knockdown; Stra8, stimulated by retinoic acid 8; DDX4, DEAD-box helicase 4; TALEN, transcription activator-like effector nuclease; TM3, mouse Leydig cell; TM4, mouse testicular Sertoli cell; hCG, human chorionic gonadotropin.

**Table 3:** Role of m^6^A and its regulators in folliculogenesis and oogenesis

Period of occurrence	Regulator	Species	m^6^A perturbation	Phenotype	mRNA biology	Ref.
Primary follicle	KIAA1429	Mouse	*Kiaa1429* KO (Zp3)	Completely infertile	Processing and alternative splicing	[97]
Oocyte		Pig	Cycloleucine	Chromosomal alignment/spindle organization	MPF signaling pathway	[82]
Oocyte at PG, PV, EV, and MV stages	Mettl3	Zebrafish	*mettl*3 KO (TALENs)	Block oocyte maturation		[23]
GV oocyte	METTL3	Mouse	*Mettl3* KD (siRNAs or morpholino)		Translation, degradation, and zygotic gene activation	[79]
GV oocyte	KIAA1429	Mouse	*Kiaa1429* KO	Reduce m^6^A transcripts and impair oocyte competency	Stability	[96]
GV oocyte and MII oocyte		*Xenopus laevis*			Transcription and translation	[78]
FGC	YTHDC2	Mouse	*Ythdc2* KD (STRA8+)	Meiotic entry and impair progression of meiosis prophase I		[60]
Postnatal oocyte	YTHDC1	Mouse	*Ythdc1* KO (DDX4, Zp3)	Lack of secondary or antral follicles	Alternative splicing, processing, and translation regulation	[105]
Oocyte	YTHDF2	Mouse	*Ythdf2* KO (Zp3)	Female-specific infertility	Transcriptome	[106]
Embryo-derived BME cell line and ovary-derived BmN4-SID1 cell line	METTL3, METTL14	Silkworm	Dot blot	Cell cycle arrest, deficiency of chromosome alignment, and segregation	Expression and translation	[92]
Follicle selection		Chicken			Wnt pathway	[81]

*Note*:  siRNA, small interfering RNA; Zp3, zona pellucida 3; PG, primary growth; PV, previtellogenic; EV, early vitellogenic; MV, mid-vitellogenic; GV, germinal vesicle; FGC, female germ cell.

Based on the current evidence, in both animals and humans, dysregulation of RNA m^6^A modification is closely related to dysspermatogenesis, leading to abnormal semen formation. Therefore, reversing this dysregulation may offer promising prospects for the treatment of infertility.

## m^6^A in embryogenesis

Two gametes combine after fertilization to form a new individual. In human embryos, gene expression begins at the one-cell stage [109]. During transcriptional silencing following fertilization, the genome is reprogramed to allow the embryo to develop into a new individual [110]. The two primary windows of epigenetic reprogramming have been demonstrated in mouse models: (1) gametes are formed when oocytes undergo DNA demethylation, and thereafter, the genome is gradually remethylated, including imprinted genes and transposon regions; and (2) after fertilization, during early embryonic development, global demethylation (except for imprinted genes) and remethylation occur to establish a genealogy [111,112]. Through the MZT process, in which the breakdown of maternal products is synchronized with zygotic genome activation (ZGA), transcriptional control is transferred to the zygote [113]. An essential MZT event in animal embryos is the excision of a subset of maternal transcripts that accumulate during oogenesis. Invertebrates and vertebrates both have a maternally encoded mRNA decay mechanism (M-decay) that is activated before ZGA, while a second pathway that requires zygotic transcription clears more mRNAs afterward (Z-decay) [114]. In Z-decay and ZGA, the RNA expression of m^6^A transcripts (transposable elements MTA and MERVL) was greater than that of their unmarked counterparts, while M-decay transcripts did not significantly differ in the m^6^A status. Most maternal RNAs degrade during oocyte maturation and fertilization during the MZT process, and the initial wave of ZGA occurs between the late one-cell and late two-cell stages [96]. m^6^A methylation is essential for maintaining embryonic stem cells (ESCs) in a ground state [85]. Recent research has suggested that thousands of mouse ESC (mESC)-specific transcripts, including long intergenic non-coding RNAs, are changed by m^6^A and this alteration can control the fate of mESCs [115].

m^6^A-mediated maternal mRNA clearance is regulated by a zygotic program [13]. The overall abundance of m^6^A continuously decreases during MZT, and the number of m^6^A transcripts gradually increases after fertilization [96] (Figure 4). The RNA-m^6^A modification landscape of human fetal tissues, however, reveals that several m^6^A peaks are present in introns and intergenic regions and that m^6^A is positively correlated with gene expression homeostasis, most likely by limiting or buffering gene expression perturbations at the post-transcriptional level [116]. During these periods of epigenetic recombination, genomic expression is highly affected by environmentally induced epigenetic defects.

The m^6^A modification dynamically changes during embryonic preimplantation in mice, and it is higher in the blastocyst stage than in the two-cell, four-cell, and eight-cell stages [117,118]. Enzyme-linked immunosorbent assay showed significantly increased levels of m^6^A-marked RNAs in female germ cells (FGCs) at embryonic day 13.5 (E13.5) and E14.5 when compared with those at E12.5 [60]. The m^6^A content is markedly enriched in early embryogenesis in *Drosophila* but declines sharply 2 h after fertilization and stays low for the remainder of embryogenesis and the early larval stages [24]. However, in pigs, m^6^A methylation continues from the zygotic stage to the blastocyst stage and suddenly increases significantly during the transition from the morula to the blastocyst [82,119]. In summary, m^6^A undergoes dynamic changes during embryonic development.

### m^6^A writers in embryogenesis

Changes in m^6^A and its regulators affect the progression of embryogenesis. Methyltransferases are highly conserved throughout evolution and are crucial for embryonic development [120]. *Mettl3* KD impacts the decay of m^6^A ZGA transcripts [96]. In *Mettl3* KO mice, nearly half of the two-cell stage embryos did not develop normally into four-cell stage cells, which impeded the MZT and ZGA processes [79]. KD of *Mettl3* and *Mettl14* in mESCs reduces m^6^A RNA methylation and results in a loss of self-renewal capacity [85]. *Mettl14* KO mice, to a large extent, showed growth retardation and morphological abnormalities and died at E8.5 [121]. KIAA1429 is a newly discovered writer, and m^6^A generated via KIAA1429 helps stabilize Z-decay mRNAs in mouse oocytes [96]. *Zc3h13* KD in mESCs significantly decreased global m^6^A level, and *Zc3h13* KO resulted in morphological changes and reduced self-renewal ability of mESCs [41]. In zebrafish, *mettl3*-null males and females have much lower rates of reproduction, and the sex ratio is impacted by the loss of *mettl3* [23]. The establishment of a gonadal structure and function depends on the development of both germline and somatic cells [43]. Regarding gonadal development, in *C*. *elegans*, METT-10 (a METTL16 homolog) promotes vulva, somatic gonad, and embryo development, ensuring the differentiation of germ cells during meiosis [10,93]. The *METTL3* and *IME4* homologs in *Drosophila melanogaster* are mainly expressed in the testicles and ovaries, and *Mettl3* is necessary for Notch signaling during oogenesis. Flies with *Ime4* loss have a fatal phenotype [122]; however, recent research has indicated that m^6^A is necessary for female-specific alternative splicing, and that *Drosophila Ime4*-null mutants are viable and fertile despite being unable to fly [22,24]. Successful blastocyst implantation is necessary for normal embryonic development after fertilization [123]. Decidua and placenta development must be closely coordinated during pregnancy to protect the fetus from maternal immune system attacks and to support fetal growth [124]. Many clinical conditions, such as preeclampsia and abortion, are associated with placental function. Changes in m^6^A modification also affect decidualization. The compromised postimplantation development of *Mettl14* KO embryos may be due to defects in epiblast differentiation [121]. In *Wtap* mutant mice, the endoderm and ectoderm of the embryo did not develop normally [40].

### m^6^A erasers in embryogenesis

*Fto* KO leads to closed chromatin in mESCs [104]. By up-regulating FLOT2, *FTO* causes granulosa cell dysfunction, which raises the possibility that FTO/FLOT2 may be involved in the pathogenesis of polycystic ovarian syndrome (PCOS) [125]. In a prior study, compared with normal individuals, the *FTO* level was lower and the m^6^A level was higher in women with spontaneous abortions [126]. Multiple abnormalities and severe developmental retardation can result from the loss of FTO function [127]. Embryos with *FTO* deletion show delayed development, and the maternal loss of *FTO* severely impedes decidua formation and embryo generation [104]. *ALKBH5* KD in trophoblasts promotes trophoblast invasion. In contrast, the overexpression of *ALKBH5* inhibits cell invasion [128].

### m^6^A readers in embryogenesis

In mice, preimplantation embryos, MII oocytes, and postnatal oocytes all have high levels of YTHDC1, while GV-stage oocytes have low levels [105]. *Ythdc1* cKO mESCs exhibit markedly reduced proliferation rates [129]. Compared with those at E12.5, *Ythdc2* expression was considerably higher in FGCs at E13.5 and E14.5. Cycloleucine-induced m^6^A inhibition or *Ythdc2* KD in FGCs prevents meiotic entrance and prophase I progression [60]. Of two studies in zebrafish, one reported that removing *ythdf2* prevented ZGA and accelerated the degradation of m^6^A-modified maternal mRNAs in zebrafish embryos [59]; the other study reported that the commencement of gastrulation, ZGA, and global maternal mRNA clearance were not impacted by the genetic deletion of the m^6^A readers Ythdf2 and Ythdf3 [13]. In summary, normal growth, sex determination, gonadal development, and zygotic gene activation are all impacted by m^6^A alteration (**Table 4**).

m^6^A modification is a dynamically changing process that plays an integral role in fertilization, embryonic development, embryo implantation, and postimplantation development. As shown by animal experiments and clinical trials, abnormalities in m^6^A modification and its modifiers lead to various development abnormalities. Understanding these processes may be helpful for drug research.

**Table 4.** Role of m^6^A and its regulators in embryonic development

Period of occurrence	Regulator	Species	m^6^A perturbation	Phenotype	mRNA biology	Refs.
ESC	YTHDC1	Mouse	*Ythdc1* KO	Reduce proliferation rate	Decay	[129]
ESC	ZC3H13	Mouse	*Zc3h13* KD	Cell morphological changes and reduced self-renewal ability	Reduction of m^6^A in the 3′ UTR	[41]
Early embryogenesis	Ythdf2	Zebrafish	*ythdf2* KO	Block late G2 phase or impair early M spermatogenesis, up-regulate maternal transcripts, and down-regulate zygotic transcripts	Stability and degradation	[59]
Embryo	Ime4	*Drosophila*	*Ime4* KO	Sex determination (control female survival）	Alternative splicing, and notch signaling	[22,24]
Embryo	METTL14	Mouse	*Mettl14* KO (px330)	Largely growth retardation and aberrant morphology	Alternative splicing and defects in epiblast differentiation	[121]
Sporulating	IME4	Yeast	Lack of a functional *IME4* gene	Severe reduction in sporulation	Splicing, stability, and translation	[95]

*Note*: ESC, embryonic stem cell; UTR, untranslated region; IME4, Inducer of MEiosis 4.

## Potential targets for m^6^A modification

Alterations in epigenetic modifications might be the focus of chemical therapeutic design and production to restore proper expression [6,130,131]. Inhibitors of m^6^A writers and erasers have been investigated. Due to the limited coverage of m^6^A modifications for reproductive health, we have summarized other aspects of m^6^A drugs.

In 1997, METTL3 was identified as a key catalyst of the m^6^A modification [34]. *METTL3* KD reduces methylation levels, accelerates apoptosis, reduces hyperplasia, and inhibits tumor growth in various cancers (acute myeloid leukemia; glioblastoma; uveal melanoma; osteosarcoma; oral, head and neck, and cutaneous squamous cell carcinoma; nasopharyngeal carcinoma; and breast, liver, bladder, gastric, prostate, lung, colorectal, pancreatic, thyroid, and ovarian cancer) [132–151]. The SAM simulator was the first METTL3 inhibitor discovered [152], but it has not been used clinically because of its poor cellular permeability and selectivity against other methyltransferases. Glioblastoma progression can be stopped by *METTL3* overexpression in tumor cells or by pharmacologically inhibiting *FTO* demethylase [153]. Exon junction complexes are m^6^A inhibitors that protect proximal exon-binding RNA in the coding sequence from methylation and regulate mRNA stability through m^6^A inhibition [154]. Exon junction complexes are thought to be part of a new family of m^6^A regulators and suppressors that largely prevent the deposition of m^6^A [154]. Research continues on their ability to modulate gene expression outcomes.

Nonspecific FTO inhibitors include the 2OG competitors *N*-oxalyl-glycine 1 and fumarate 2 [155,156], and FTO selective inhibitors include fluorescein derivatives FL2-DZ [157], a kind of compound [half-maximal inhibitory concentration (IC50) = 0.81 mM] [158], and entacapone [155]. Rhubaric acid-3 inhibits both the FTO and ALKB subfamilies [156,159]. A compound (IC50 = 8.7 mM) reduced *FTO* expression and showed potential usefulness in the treatment of epilepsy [160]. The FTO selective inhibitor MO-I-500 inhibited the survival and/or colony formation of triple-negative inflammatory breast cancer cell lines [161,162]. The FTO inhibitors R-2HG and FB23-2 can inhibit the growth of leukemia cells and slow the progression of leukemia; FB23-2 is more effective and can significantly inhibit acute myeloid leukemia progression in xenograft mice [163,164]. FTO regulates liver gluconeogenesis through the FOXO1 axis, and entacapone acts as an inhibitor of FTO, regulating fasting blood glucose through its direct effect on the liver FTO–FOXO1 signaling axis [165]. Studies on ALKBH5 inhibitors are limited.

To date, both authorized medications and random substances with known activity have been investigated as m^6^A regulators [166]. The gut microbiome has a profound impact on a variety of elements of human health and disease, causing a variety of host reactions, including significant genetic changes. By lowering m^6^A levels in the intestinal mucosa of weaned piglets, resveratrol and curcumin could successfully enhance growth performance and maintain the integrity of the intestinal mucosa [167]. Thus, microorganisms can also affect the m^6^A modification level of mRNA in host tissues. In a clinical trial, the butyric acid levels in the digestive tracts of obese women with PCOS were lower than those in normal controls. Butyric acid reduces the expression of inflammatory cytokines by suppressing the expression of *METTL3*, increasing ovarian function, and lowering the expression of local inflammatory factors in the ovaries [168]. The degree of global nucleic acid methylation, which includes DNA methylation and RNA m^6^A modification, was also lowered by exogenous vitamin C supplementation in immature swine Sertoli cells, increasing reproduction [169]. The m^6^A mutation in viral RNA helps viruses evade innate immunity [170]. Consequently, the application of m^6^A modification in the therapy of particular disorders is possible. These findings offer a fresh viewpoint for diagnosing and treating reproductive illnesses such as infertility.

## Epigenetics and reproduction

With the rapid development of assisted reproductive technology, epigenetic research has helped promote the treatment of specific abnormalities; however, assisted reproductive technology induces epigenetic changes that could impair the development of fertilized mouse oocytes [171]. For example, during spermatogenesis, specific cells can be treated through epigenetic intervention. Sperm DNA is methylated differently in several maternal and paternal imprinted areas and shows a unique global methylation pattern [172]. During the gametogenesis and peri-implantation stages, reprogramming epigenome and imprinted loci, particularly imprinted loci, is important for maintaining the correct genetic pattern [172]. During germ cell development, histone deacetylation in oocyte maturation, energy metabolism, fertilization, mitochondrial function, genomic imprinting, and embryo genome activation are all involved in epigenetic inheritance [173]. When hormone stimulation fails to produce mature MII oocytes, *in vitro* maturation can be considered. Epigenetic regulation, such as histone acetylation and methylation, determines the quality of *in vitro* maturation [174]. As shown by genome sequencing technology, newborns conceived through assisted reproductive technology exhibit differences in methylation genes compared with those conceived naturally [175,176]. *ALKBH5* mRNA expression in the chorionic tissue of patients with recurrent abortion is significantly increased, impairing the function of the trophoblastic layer [128]. Genetic susceptibility to PCOS is not only associated with alleles, but is also influenced by epigenetic changes [177]. Epigenetic aberrations have also been found in the ectopic endometrium of patients with endometriosis, and an understanding of the epigenome of the ectopic endometrium is key to understanding the genetic dysregulation and coordination that impair endometrial tolerance [178,179].

## Future perspectives

Changes in genetic material can lead to the development of many diseases. The secrets of epigenetics have gradually been revealed, explaining phenomena that heredity cannot explain. The transmission of epigenetic modifications is relatively stable during cell proliferation [180]. Drugs that can modify epigenetic processes, could be used to treat certain diseases. With advancements in epigenetic research, we may understand how the environment impacts fertility and devise mechanisms to prevent infertility. Studies have been conducted to derive male germ cells from pluripotent cells, and research is progressing to obtain oocytes from stem cells [181,182]. Since many procedures undertaken during *in vitro* fertilization (IVF) are conducted during the critical period of epigenetic recombination, including the removal of existing epigenetic modifications and replacement with new modifications in the somatic cell tissues of gametes and embryos [183], IVF may cause epigenetic alterations. Therefore, it is important to gain an in-depth understanding of the epigenetic changes occurring during IVF to reduce long-term complications in children.

Many challenges still lie ahead. The mechanisms of some diseases are not clear, and a deeper understanding of these mechanisms is critical to the treatment of the disease. Research in other areas of study such as organoids, pluripotent stem cell differentiation, and synthetic biology [184], will further enhance our approach to understanding the mechanisms of infertility. Further in-depth research on the genetics and epigenetics of infertility can provide both short-term and long-term benefits. We will be able to improve the outcomes of pregnancy and the long-term health of the population through epigenetic treatment and promote the development of personalized drugs for fertility treatment. Creating *in vitro* models for spermatogenesis and oogenesis and investigating whether novel medications have an impact on gametogenesis are the best immediate focuses of clinical research [172].

## Conclusion

RNA methylation is an important epigenetic modification. The dynamic changes in RNA m^6^A modification are caused by methyltransferases, demethylases, and m^6^A-binding proteins, and they affect various physiological and pathological processes, including spermatogenesis, oogenesis, embryogenesis, placental function, and human reproductive system diseases. Reproduction is one of the most strictly regulated key processes in the mammalian life cycle, and reproductive health is essential for human reproduction. Animal studies have shown that RNA m^6^A modification plays a crucial role in gametogenesis, embryonic development, and placental function. The theoretical foundation of germ cell development can be strengthened by understanding the functions of m^6^A regulators in gametogenesis and embryonic development, which could also help us uncover novel causes of infertility. As another important regulatory layer, RNA m^6^A modification in somatic cells of the reproductive system deserves extensive research. The regulation of RNA m^6^A modification imbalance can be used to restore function in the reproductive system and may help determine its core clinical value in infertility. m^6^A modification and its regulators can be targeted by drugs to treat certain diseases. Although this discovery has important theoretical and practical significance, it remains within the realm of basic research and has not yet undergone clinical translation.

## CRediT author statement

**Yujie Wang:** Writing – original draft, Visualization. **Chen Yang:** Writing – review & editing. **Hanxiao Sun:** Writing – review & editing. **Hui Jiang:** Visualization. **Pin Zhang:** Supervision. **Yue Huang:** Supervision. **Zhenran Liu:** Supervision. **Yaru Yu:** Supervision. **Zuying Xu:** Supervision, Funding acquisition. **Huifen Xiang:** Funding acquisition, Writing – review & editing. **Chengqi Yi:** Conceptualization, Writing – review & editing. All authors have read and approved the final manuscript.

## Competing interests

The authors have declared no competing interests.
